# Unveiling the Rarity: A Case of a Mixed Neuroendocrine-Non-Neuroendocrine Tumor of the Gastroesophageal Junction

**DOI:** 10.7759/cureus.63141

**Published:** 2024-06-25

**Authors:** Faris Alamin, Hannah Sage, Isabel Torres, Jignesh Parikh, Vania Zayat

**Affiliations:** 1 Internal Medicine, University of Central Florida/HCA Osceola Hospital, Orlando, USA; 2 Pathology, University of Central Florida College of Medicine, Orlando, USA; 3 Pathology, Orlando Veterans Affairs Medical Center, Orlando, USA

**Keywords:** esophageal neuroendocrine, mixed neuroendocrine-non-neuroendocrine neoplasm, acute gi bleed, minens, gastroesophageal junction (gej)

## Abstract

Mixed neuroendocrine-non-neuroendocrine neoplasms (MiNENs) are a rare group of heterogeneous tumors, consisting of an endocrine and a nonendocrine component, which can develop throughout the gastrointestinal (GI) tract. This case presents a 70-year-old man with a complex medical history who initially presented with an upper GI bleed. After being stabilized, he underwent an esophagogastroduodenoscopy (EGD) that revealed a suspicious gastroesophageal junction (GEJ) mass. Histopathological studies paired with immunohistochemical investigations of the mass confirmed the rare diagnosis of MiNENs. He then underwent an endoscopic submucosal dissection (ESD) with subsequent chemotherapy and adjunct radiotherapy, with no recurrence noted on post-treatment surveillance. This case highlights the need for an EGD, histopathological examination, and immunohistochemical staining for detecting the underlying etiology of an upper GI bleed.

## Introduction

Mixed tumors were first described by Cordier in 1924 as a GI neoplasia with an adenocarcinoma and a neuroendocrine component [1]. However, since then, there has been significant controversy and changes in the development of histopathologic criteria [2]. This type of tumor is distinguished by a mixture of neuroendocrine and non-neuroendocrine components, each accounting for > 30% of the tumor. Previously described as mixed adeno-neuroendocrine carcinoma (MANEC), with current nomenclature, 2019 WHO digestive system classification and 2022 neuroendocrine classification renamed to mixed neuroendocrine-non-neuroendocrine (MiNEN) instead, to account for morphological heterogeneity of the disease [3,4]. Generally, gastrointestinal and pancreatic MiNENs have an estimated 0.735 cases per 1,000,000 person-years in 2017 [5]. Moreover, among GI MiNEN, esophageal/GEJ MiNENs are exceedingly rare, with a mere 10 reported cases in 2019 [6]. Herein, a case of GEJ MiNEN is discussed.

## Case presentation

**Figure 1:**
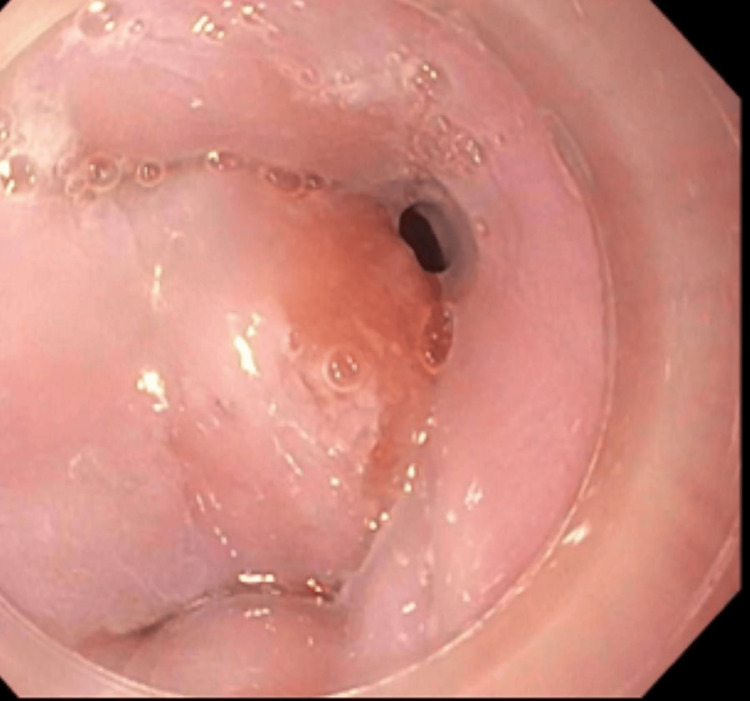
Endoscopic view of the gastroesophageal junction mass Images reproduced with patient permission.

**Figure 2:**
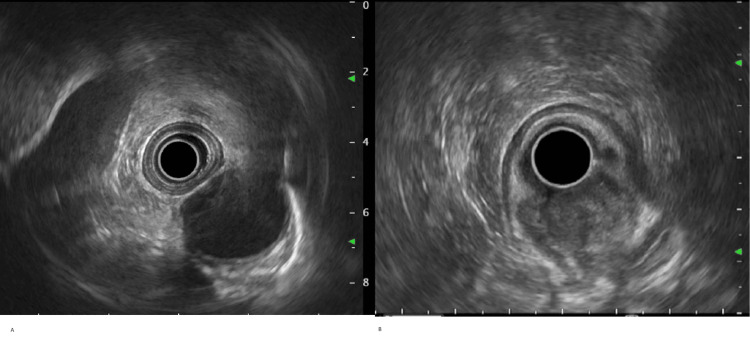
Endoscopic ultrasound (A) There was no sign of significant endosonographic abnormality in the proximal and middle esophagus. (B) A hypoechoic oval mass was identified endosonographically, in the gastroesophageal junction, extending to cardia of the stomach. This was staged T1sm N0 Mx by endosonographic criteria. There was sonographic evidence suggesting invasion into the submucosa (layer 3). Images reproduced with patient permission.

**Figure 3:**
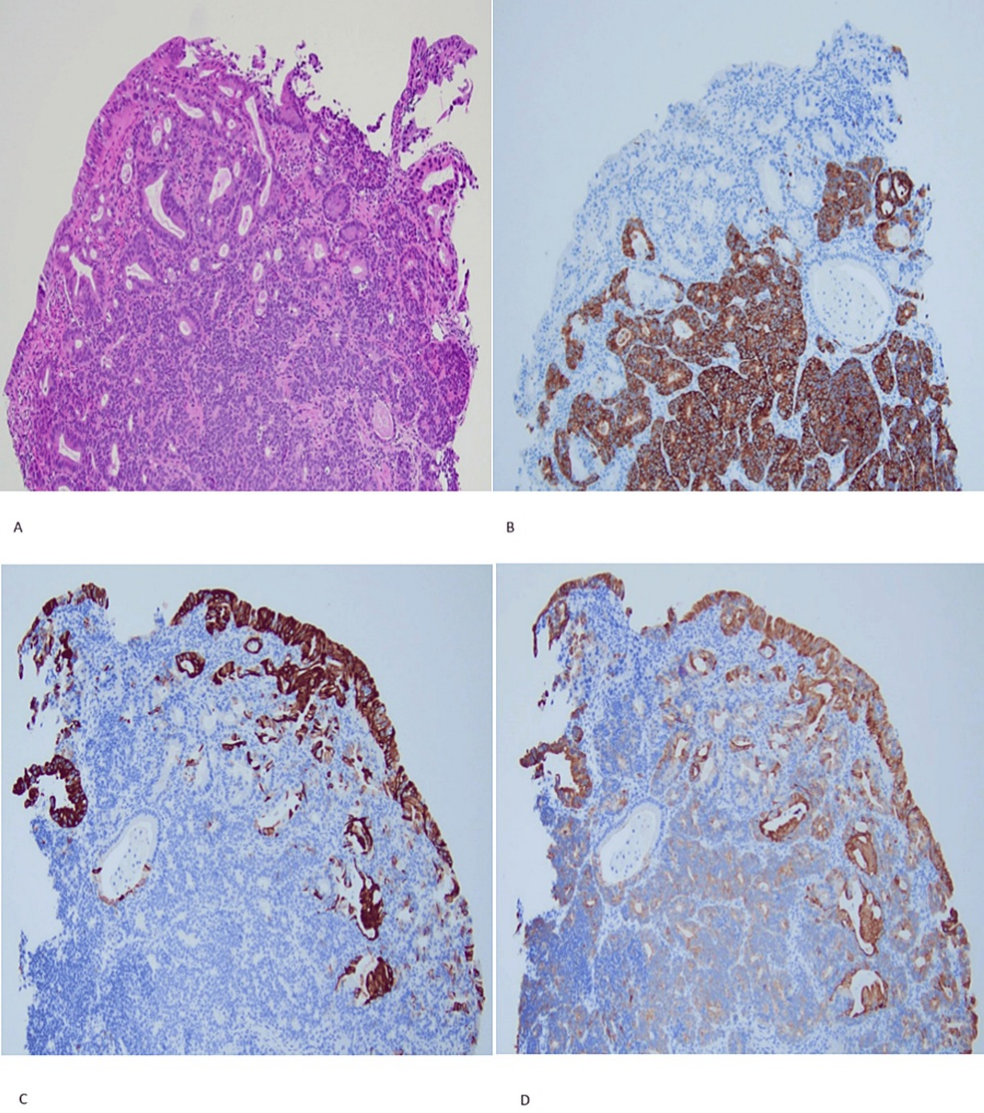
Pathology microphotographs (A) The H&E-stained section shows mixed adenocarcinoma and neuroendocrine carcinoma. The adenocarcinoma component shows well-differentiated glands. Neuroendocrine carcinoma cells show hyperchromatic nuclei, scant cytoplasm, and some rosette formation. (B) CK7, CK20, CD56, and synaptophysin-stained section. The neuroendocrine carcinoma component is positive for synaptophysin, CD56, and CK7 (weak) and negative for CK20 immunostains. (C) CK7, CK20, CD56, and synaptophysin-stained section. The adenocarcinoma component is positive for CK7 and negative for synaptophysin and CD56 immunostains. (D) CK7, CK20, CD56, and synaptophysin-stained section. The adenocarcinoma component is positive for CK20 and negative for synaptophysin and CD56 immunostains. All micrographs shown magnified X20. Images reproduced with patient permission.

A 70-year-old man with a history of coronary artery disease status post coronary artery bypass graft (CABG), paroxysmal atrial fibrillation, and pleural plaques secondary to remote asbestos exposure, initially presented to the emergency with hematemesis and a syncopal episode. The patient was taking rivaroxaban, amiodarone, and rosuvastatin. The patient was found to be severely anemic with a hemoglobin of 8 g/dl, was appropriately stabilized, and given a packed red blood cell (RBC) transfusion. As part of the workup of the GI bleed, the patient underwent an EGD, which revealed a highly suspicious, likely malignant, 12 mm in thickness mass, at the gastroesophageal junction that was biopsied. There was sonographic evidence suggesting invasion into the submucosa (layer 3). A normal first and second portion of the duodenum with normal gastric body and antrum were noted. The rivaroxaban was stopped and the patient was discharged from the hospital with a planned close follow-up. A few days later, the patient underwent a repeat EGD (Figure 1) and endoscopic ultrasound (Figure 2) where the mass was visualized and staged at T1N0 M0 by endosonographic criteria. Complete removal of the mass involving a 30 mm area from the GEJ extending to cardia was accomplished with a hybrid endoscopic submucosal dissection (ESD) technique. Histological examination of the mass showed areas composed of classic adenocarcinoma morphology and areas that are morphologically and immunophenotypically showing neuroendocrine differentiation. The adenocarcinoma areas were positive for CK7, CK20, CDX2, polyclonal CEA, and focal CD117 and negative for synaptophysin and CD56 (Figure 3). However, the neuroendocrine differentiation areas were positive for synaptophysin, CD56, CDX2, and CK7, and negative for CK20 (Figure 3). Deep and peripheral margins were positive. American Joint Committee On Cancer (AJCC) stage was diagnosed as T1bN0M0. The histological and immunophenotypical studies confirmed the diagnosis of MiNENs at the GEJ. Further molecular studies yielded an unclear HER2/neu with subsequent negative fluorescence in situ hybridization (FISH) testing.

The patient was evaluated by a multidisciplinary team including medical oncology, surgical oncology, and radiation oncology. They deemed the patient as a high-risk candidate for surgical treatment given significant comorbidities and were treated with an eight-week regimen of carboplatin and paclitaxel along with radiation therapy. The patient has been followed up for a year without any signs of recurrence to date.

## Discussion

Mixed neuroendocrine non-neuroendocrine neoplasms (MiNENs) are a rare type of malignancy arising anywhere along the gastroenteropancreatic tract and are characterized by histologically evident neuroendocrine and non-neuroendocrine components each consisting of 30% of the total tumor [3,4,7]. While limited, available data indicates that these tumors occurred in a male predominance and with a median age of 64 years old at the time of diagnosis [7]. Interestingly, it was found that between 2004 to 2016, the age-adjusted incidence of MiNEN increased from 0.01/100000 person-years to 0.07/100000 person-years, which may be attributed to improved diagnostic techniques and clinical recognition [7,8]. In a study comparing multiple case reports of individuals with MiNENs, the most common primary tumor sites were found to be the stomach, biliary tract, and colon [7]. Diagnosis is made via biopsy with histologic review and immunohistochemical staining [4]. Esophageal MiNENs comprise a small percentage of all MiNENs, with one study showing them to account for 5.9%-15.9% of cases [7]. Most cases of esophageal MiNEN consist of neuroendocrine carcinoma (NEC) and squamous cell carcinoma (SCC), although adenocarcinoma has been rarely reported as the non-neuroendocrine component [7]. Taken together, this shows that our case of MiNEN at the gastroesophageal junction consisting of neuroendocrine and adenocarcinoma components is particularly exceedingly rare.

According to the 2022 WHO classification of neuroendocrine neoplasms, a mixed neuroendocrine-non-neuroendocrine neoplasm is classified as a neoplasm in which they are neuroendocrine and non-neuroendocrine components that are each able to be recognized morphologically and immunohistochemically [4]. Macroscopically, the tumors usually resemble the non-neuroendocrine component [5]. The site of the tumor determines what the non-neuroendocrine component can be [4]. Ninety-two percent (92%) of the time, the non-neuroendocrine portion of the tumor is adenocarcinoma of the underlying organ; in the esophagus, squamous cell carcinoma is the most common non-neuroendocrine component [2,7]. Histologically, adenocarcinoma of the GEJ can present as invasive gastric glands, invasive intestinal glands arising from intestinal metaplasia, or a combination of these [9]. Adenocarcinomas will stain positively for carcinoembryonic antigen, CA 19-9, and cytokeratins 7, 19, and AE 1/3 [7]. This is consistent with the histological examination and immunostaining of the adenocarcinoma component of our case. Mucin will also stain positively with periodic acid Schiff [10]. Esophageal squamous cell carcinoma is defined by an invasion of neoplastic squamous cells into the lamina propria and deeper [11]. It stains positively for CK5/6 and p63 [11]. The neuroendocrine component can be classified as a neuroendocrine tumor (NET) or neuroendocrine carcinoma (NEC) depending on the differentiation [4]. NETs retain more morphologic and immunohistochemical features of normal neuroendocrine cells such as gland-like structures, uniform cells with granular cytoplasm, round nuclei containing finely granular chromatin, and inconspicuous nucleoli [4]. In comparison, NECs are poorly differentiated and have severe atypia and altered molecular profiles [4]. NETs are graded on a three-tiered scale and NECs are all high-grade tumors that can be further classified into small or large cell types [4]. In small cell NECs (SCNECs), the tumors demonstrate diffuse or nested small cells with scant cytoplasm, granular chromatin, and small nucleoli [4]. Large-cell NECs (LCNECs) are composed of larger cells with eosinophilic cytoplasm, vesicular nuclei with prominent nucleoli, and large areas of necrosis [4]. In most MiNENs, the neuroendocrine element is an NEC [4]. To confirm the presence of a neuroendocrine component, immunohistochemical staining is required [4]. Markers include insulinoma-associated protein 1 (INSM1), chromogranin A, and synaptophysin [4]. The WHO recommends staining for both INSM1 and synaptophysin, as it will identify most NECs and NETs [4]. In our case, the areas that histologically appeared to be of neuroendocrine differentiation were positive for synaptophysin, further supporting an NEC or NET. Chromogranin A has been negative in some NECs [4]. A case report of a MINEN in the GE junction showed strong staining for synaptophysin and chromogranin in the neuroendocrine component and stained positively with periodic acid Schiff stain in the non-neuroendocrine adenocarcinoma component [12]. In a MiNEN with a NET component, Ki67 is required for grading [4]. Tumors with an NEC component are more aggressive than those with a NET component [4]. Prognosis in NEC tumors depends on the behavior of the NEC component, whereas prognosis in NET tumors depends on the behavior of the non-neuroendocrine component [4]. Nevertheless, both components of the tumor need to be graded separately to determine treatment [4].

The etiology of gastrointestinal MiNENs remains unclear; however, it has been proposed to be associated with longstanding inflammatory conditions such as Crohn’s disease [13,14]. The development of MiNENs appears to have a clonal origin from a cell with the capability for differentiation into both components. Studies have shown that both the non-neuroendocrine and neuroendocrine components of tumors share genetic mutations and loss of heterozygosity at multiple loci at higher frequencies than mutations in one component, which supports a possible clonal origin [7]. Molecularly, NETs and NECs have different mutations. NETs have epigenetic mutations that drive tumor development, whereas TP53 and RB1 mutations typify NECs [4]. In SCNECs, TP53 and RB1 inactivation is usually biallelic [4].

The differential diagnosis of MINEN includes paraganglioma [4]. Paragangliomas are tumors that mostly consist of chromaffin cells that are organized in a honeycomb pattern with well-circumscribed nests surrounded by supratentorial cells [15]. Paraganglioma can be distinguished by immunohistochemical staining for GATA3 and stains for enzymes that synthesize catecholamines [4]. Immunohistochemical staining should be done to differentiate between a pure adenocarcinoma, a pure neuroendocrine tumor, and a MiNEN.

The prognosis for MiNEN can be estimated by determining the intermediate between the pure neuroendocrine and pure non-neuroendocrine components of the tumor [16]. In a case series of 40 patients with an esophageal poorly differentiated neuroendocrine carcinoma (PDNEC), it was found that MiNENs were less frequently metastatic than pure PDNEC, and overall survival was increased [17]. Furthermore, consideration should be given to the location of the tumor and the spread of the disease [16]. In a retrospective case series, it was found that tumors located in the upper GI tract were more commonly associated with localized disease compared with more advanced disease at diagnosis (52.1% vs 23.1%, respectively) [8]. Advanced disease includes metastasis to regional lymph nodes and the liver [18]. This finding is consistent with our case, as the patient has been without signs of recurrence to this date, which was one year after treatment.

In general, low-grade MiNEN should be treated with surgical resection of localized tumors and additional treatment for the type of NET if metastasis is present [19]. For higher-grade tumors, surgical resection is recommended along with chemotherapy and/or radiation depending on the more prominent component [19]. The WHO-recommended adjuvant chemotherapy for NEC is cisplatin or carboplatin with etoposide [19]. Our patient was treated with a similar regimen and radiation without surgery due to potential risks from comorbidities.

## Conclusions

The presented case of MiNENs arising in the gastroesophageal junction illuminates a rare and intriguing finding at this given site, which challenges conventional understanding within the field of histological diagnoses and esophageal malignancy treatment modalities. This unique finding not only highlights the importance of the need for an EGD, histopathological examination, and immunohistochemistry with meticulous observation. In addition, emphasis must be on further exploration into similar occurrences. As we delve deeper into understanding this rarity, it opens avenues for future research, encouraging a broader perspective in addressing rare malignant histological occurrences and advancing the frontiers of medical knowledge on the respective therapeutic approaches.
